# Mucosal Leishmaniasis: An Underestimated Presentation of a Neglected Disease

**DOI:** 10.1155/2013/805108

**Published:** 2013-06-18

**Authors:** Alessio Strazzulla, Salvatore Cocuzza, Marilia Rita Pinzone, Maria Concetta Postorino, Stefano Cosentino, Agostino Serra, Bruno Cacopardo, Giuseppe Nunnari

**Affiliations:** ^1^Division of Infectious Diseases, Department of Clinical and Molecular Biomedicine, Garibaldi Nesima Hospital, University of Catania, 95125 Catania, Italy; ^2^Division of Otorhinolaryngology, Department of Medical-Surgical Specialties, Policlinico-Vittorio Emanuele Hospital, University of Catania, 95125 Catania, Italy; ^3^Division of Pathology and Experimental Microbiology, Elie Metchnikoff Department, University of Messina, 98125 Messina, Italy

## Abstract

We present a review of current knowledge about mucosal leishmaniasis (ML). Although involvement of mucous membranes is classically admitted in New World leishmaniasis, particularly occurring in infection by *Leishmania (L.) braziliensis* species complex, ML is also a possible presentation of Old World leishmaniasis, in either *L. donovani* or *L. major* species complex infections. Thus, ML has to be considered not only as a Latin American disease but as an Old and New World disease. We describe ML epidemiology, pathogenesis, clinics, diagnosis, and therapy. Considering both its highly disfiguring lesions and its possible lethal outcome, ML should not be underestimated by physicians. Moreover, leishmaniasis is expected to increase its burden in many countries as sandfly vector distribution is widespreading towards non-endemic areas. Finally, the lack of clear understanding of ML pathogenesis and the absence of effective human vaccines strongly claim for more research.

## 1. Introduction

Leishmaniasis is a vector-borne disease caused by *Leishmania* protozoa (order Kinetoplastida), which is transmitted by sandflies of *Phlebotomus* and *Lutzomyia* species [1]. 1.6 millions of new cases per year are estimated to occur worldwide, but only 600,000 are recorded [2]. Moreover, leishmaniasis is estimated to affect about 12 million people in 88 countries in four continents (Africa, Americas, Asia, and Europe) [2, 3].

Classically, according to geographical criteria, leishmaniasis has been divided in two main syndromes: Old World and New World leishmaniasis. Old World leishmaniasis includes two clinical presentations: cutaneous leishmaniasis (CL), which is confined to skin, and visceral leishmaniasis (VL), which involves bloodstream and inner organs. New World leishmaniasis clinical presentations are CL and mucocutaneous leishmaniasis (MCL), which involves mucous membranes in addition to the skin [2, 3].

However, nowadays, new terms are used to describe clinical presentations of leishmaniasis. The term mucosal leishmaniasis (ML) indicates the involvement of mucosal tissues by *Leishmania* spp. In particular, ML involves mucous membranes of upper respiratory tract (from inner nostril wall to larynx) and oral cavity [2]. Typically, ML manifests from days to years after CL. This is the classic MCL or espundia [2, 4]. Together with CL and diffuse cutaneous leishmaniasis (DCL), MCL is one of the typical presentations of leishmaniasis in South America, characterizing the so-called american tegumentary leishmaniasis (ATL) [5]. MCL is typically a consequence of infection by New World *Leishmania* species, as *L*. *braziliensis*, *L*. *panamensis*, *L*. *amazonensis,* and *L*. *guyanensis* [5, 6].

Outside the South American continent, ML can exist with different and less defined clinical presentations than MCL. Indeed, ML can be either accompanied or preceded by cutaneous or VL. Anyway, mucosal involvement may be the first and only documentable pathological condition due to *Leishmania*. Generally, these clinical presentations are caused by *Leishmania donovani* complex species, commonly *L*. *infantum* [4]. 

Because of its heterogeneous way of presentation, ML is often confused and underestimated by clinicians and scientists, especially outside the South American continent. Our purpose is to sum up the clinical, epidemiologic, diagnostic, and therapeutic aspects of ML.

## 2. Epidemiology

Mucosal leishmaniasis cases have been reported in South America, Asia, Europe, and Africa.

Latin America represents the most important endemic area of ML, particularly in the region of Amazon. Indeed, its frequency ranges from 0.4% in Southern regions of Brazil to 20% in Bolivia [6]. Actually, from 1988 to 2008, 52 cases with mucosal involvement were registered in the Brazilian state of Bahia, over a court of 1209 patients with ATL [7]. In Guajara-Mirim (Brazil), from 2000 to 2003, among 170 cases of ATL, 9% presented mucosal involvement [8]. From 1982 to 2003, among 100 cases of *Leishmania* infection observed among Brazilian HIV-1 infected patients, 68% had mucosal involvement [9]. In Bolivia, which is known to have the highest incidence of ATL (33 cases/100,000 inhabitants) [10], according to data from the MHNPLC (Ministry of Health National Program of Leishmaniasis Control), from 1983 to 2006, 4619 cases of ML have been described [11]. 

In the rest of the world, ML is generally less suspected and consequently less reported. In France, according to the National Reference Center, from 1999 to 2007, 2.3% of total cases of leishmaniasis were ML [12]. In Europe, cases of ML were described in Italy, France, Spain, Malta, Portugal, Holland, the United Kingdom, and Austria [4, 12, 13]. Tunisia also presented cases of ML. Outside the Mediterranean area, ML was reported in India, Sri-Lanka, Pakistan, Iran, Saudi Arabia, and Sudan [14–26]. ML is increasingly seen in travelers [27].

## 3. Etiology

Twenty-one *Leishmania* species have been identified as human pathogens. They are systematically classified in four complexes. Two New World *Leishmania* species complexes are constituted by *L. mexicana* complex (containing also *L*. *amazonensis*) and *L*. *braziliensis* complex (containing *L*. *panamensis* and *L*. *guyanensis*), whereas two Old World *Leishmania* complexes are *L*. *major* complex (containing *L*. *tropica*) and *L*. *donovani* complex (containing also *L*. *infantum*, even known as *L*. *chagasi*) [3, 28]. *Leishmania* species of all major complexes may be responsible for ML over the world.

As the major causative agent of ATL, *L*. *braziliensis* is responsible for the highest number of ML cases in the New World. Other members of the *L*. *braziliensis* complex, such as *L*. *panamensis* and *L*. *guyanensis*, have been associated with ML [29, 30]. *L*. *amazonensis* may also cause ML [6–11].

In the Old World, particularly in the Mediterranean basin, *L*. *infantum* has been associated with ML [4, 12, 13]. Outside Europe, ML due to *L*. *infantum* was reported in Tunisia [14] and Iran [24]. Mucosal involvement also occurred during infection with *L*. *donovani* in India, Sudan, and Sri Lanka [4, 13, 15–17, 22, 24].

*L*. *tropica* and *L*. *major* may cause ML in the Old World. Interestingly, their coinfection during ML has been reported in Iran [18]. ML due to *L*. *major* or *L*. *tropica* has been described in Tunisia, Pakistan, Saudi Arabia, and Iran [19–21, 23, 25].

## 4. Ecology

Leishmaniasis is transmitted by approximately 30 species of Phlebotomine sandflies, included into two different genera, *Lutzomyia* (*Lu*.) and *Phlebotomus* (*P*.) [3].

The life cycle of *Leishmania* presents two stages: firstly, a promastigote (extracellular and flagellated) stage, which takes place in the vector gut; then, after injection into a mammalian host by the byte of a female sandfly, *Leishmania* body shifts into the amastigote stage, with amastigotes surviving inside phagocyte cells (mainly macrophages) [3].

Thus, it is not surprising to find out that ML distribution reflects the natural diffusion of *Leishmania* vectors either in South America or in the Mediterranean basin [10, 12]. Interestingly, either in South America or Europe, severe ecological mutations are likely to deeply change current leishmaniasis scenario.

As a matter of fact, in Latin America, leishmaniasis has always been related to the forest habitat (pluvial rainforest and agricultural fields close to the forest), where reservoir mammals live in close association with humans. Nowadays, leishmaniasis is largely moving towards domestic habitats, as a direct consequence of the spreading of *Leishmania* vectors to urbanized areas, especially outskirts [10, 11]. The increasing number of long-distance travels and the spreading of vectors towards currently not endemic areas are likely to cause a marked rise in the incidence of leishmaniasis also in Europe [31, 32].

## 5. Pathogenesis

The immunopathogenesis of ML is complex and still partially unknown [31]. 

Sandflies play an important role in the development of leishmaniasis, not only as vectors but also as active players. In an animal model of ATL, it has been shown that contemporary inoculation of parasites (*L*. *braziliensis* and *L*. *amazonensis*) and vector saliva exacerbated infection, thus pointing out that vector saliva contains substances with a potential immune regulatory role. Furthermore, uninfected sandfly bites seem generally able to produce protection to *Leishmania* spp. (especially to either *L*. *amazonensis* or *L*. *infantum*), whereas *Lu*. *intermedia* bites may enhance *L*. *braziliensis* infection. For Old World species, a protective role is possible in case of *P*. *papatasi* and *L*. *major* infection, whereas *Lu*. *longipalpis* saliva may protect versus the development of VL [28]. 

Another interesting point is the high variability of *Leishmania* species and strains causing ML. Indeed, ML has been associated with *L*. *infantum* zymodemes MON-1 (viscerotropic), MON-24, MON-27, MON-80, or MON-111 (viscerotropic and dermotropic) [12]. Similarly, profound genetic differences have been reported between *L*. *braziliensis* strains causing CL and ML [33]. Interestingly, it has been hypothesized that some *Leishmania* strains (particularly *L*. *infantum* strains involved in isolated ML) have developed different resistance to high/low temperature, acquiring the capability to live electively in mucous membranes [4, 13]. 

Some studies have evaluated the role of *Leishmania* RNA Viruses (LRVs) in the pathogenesis of ML. These viruses can infect American *Leishmania* species, notably *L*. *braziliensis* and *L*. *guyanensis*. Of note, LRVs have been detected in lesions from ML, either in hamster models and human samples, whereas LRVs were absent or scarcely present in CL lesions [34]. Furthermore, LRV-1 has been associated with high levels of cytokines and chemokines and with the presence of destructive metastatic lesions during *L*. *guyanensis* infection [35].

Host adaptive immunity is decisive in addressing *Leishmania* infection towards ML or CL pathways. Simplifying, interleukin- (IL-) 10 levels are equally high in ML and CL, whereas interferon- (IFN-) *γ* and tumor necrosis factor-*α* production are higher in ML than in CL. In ML, CD4+ T cells are the major source of cytokines; production of anti-inflammatory molecules from monocytes is reduced and either CD8+ T cells or natural killer cells contribute to tissue destruction in late stages. Symptomatic patients have lower IFN-*γ* and higher IL-10 levels than asymptomatic ML patients, that is, patients potentially exposed to *Leishmania* infection since they come from endemic areas with epidemiological evidence of parasite transmission [33]. Furthermore, it has been proved that ML develops in the presence of insufficient or misled immune response in CL early stages [36]. Nevertheless, current experimental evidences show that ML is associated with uncontrolled and self-feeding inflammation [33]. Unfortunately, there is a lack of experimental models for ML caused by *Leishmania* species other than *L. braziliensis* complex.

Immunodeficiency enhances the development of ML. In ATL, HIV/*Leishmania* coinfected patients presented higher rates of ML (46.7–68%) than *Leishmania* mono-infected patients (1.5%) [9]. HIV infection negatively affects CL development and outcome. Indeed, it favors atypical and disseminated localizations, enhancing T helper- (Th-) 2 responses and limiting Th-1 [37]. Either ML by *L*. *braziliensis* (and *L*. *guyanensis*) or ML by *L*. *infantum* seems to be potentiated by low CD4+ T-cell count [9, 12]. Moreover, local mucosal immunodeficiency may contribute to the development of leishmaniasis. Indeed, it has been supposed that several mucosal immunosuppressive factors, as tobacco smoke, corticosteroid therapy (systemic and inhaled) and upper respiratory diseases, may facilitate ML [4, 12, 13]. 

Another controversial point is the way used by protozoa to reach mucous membranes. Indeed, there are at least three different ways. Firstly, ML caused by *L. major* is generally a consequence of direct extension of contiguous facial skin lesions [14, 23]. Secondly, in *Leishmania braziliensis* complex ML, the involvement of mucous membranes is generally subsequent to skin ulcers that, differently from *L*. *major *ML, can develop also in different sites, such as arms, trunk, and legs, suggesting a lymphatic/haematogenous diffusion of parasites [6, 8, 38]. However, it is not clear whether ML is due to reactivation of latent parasites which had previously caused CL or to a new infection [8]. Similarly, diffusion via bloodstream appears the most likely route in case of *L*. *infantum* ML, which is not usually preceded by any skin lesions [12]. The description of isolated ML in immunocompetent patients may suggest that mucosal localization is not the result of uncontrolled progression of the disease, but it may reflect the attempt of the immune system to confine it [13]. Thirdly, direct mucosal injection of parasites through sandfly bite appears possible for oral and nasal localization [19], whereas it seems unrealistic in case of isolated leishmaniasis of inner sites (i.e., larynx) [4, 12, 13].

Finally, ML is more common among adult men than among adult women and children in both Old and New World. It could be related to occupational exposure factors, according to the fact that rural workers are generally males [7, 8]. Anyway, even endocrinal factors could play a role in the pathogenesis of ML [12]. 

## 6. Clinical Aspects

Clinical presentation of ML can significantly vary, according to the heterogeneity of pathogens and complexity of pathogenesis.

Classical mucosal lesions occurring during ATL are highly destructive, severely disfiguring, and potentially deadly. Typical lesions are ulcerated and often lead to septum perforation. Cutaneous lesions precede ML in 5–20% of cases [39]. These lesions can be clinically manifested or healed, from days to decades before mucosal involvement [6, 38]. High number of cutaneous lesions and mistreatment are risk factors for evolution towards ML [8]. Notwithstanding, isolated ML due to *L. braziliensis* complex species has been described, with a frequency of 17-18% among patients with mucosal involvement [7, 8]. Upper respiratory tract, nose, and oral cavity are the most frequent initial sites of ML lesions, whereas pharyngeal and laryngeal involvements usually come later, as a consequence of disease progression. Initially, ML clinically starts with unspecific symptoms of inflammation (i.e., nasal congestion, erythema, edema, serous rhinorrhea, and epistaxis), which progressively worsen (insurgence of dysphagia and dysphonia), as a consequence of deterioration of soft tissues of nose, mouth, and throat and progression towards lower respiratory tract [2, 6, 38–40]. In a case report, MCL was characterized by ocular involvement, presenting with exophthalmos [41]. Because of unspecific clinical findings, several infectious diseases can enter the differential diagnosis, such as mycobacteria infections, schistosomiasis, blastomycosis, and leprosy [6].

*L*. *major* and *L*. *tropica* can also cause ML. Generally, it is accepted that mucosal involvement is the consequence of a progression of facial skin lesions, which progressively extend to oral and nasal mucous membranes or cartilages [14, 23]. Anyway, isolated ML by *L*. *major* complex species has been reported [14, 18–21]. In ATL, mucosal lesions are less severe and destructive [20, 23], although they may be significantly disfiguring [23]. Lesions may involve the nose (endonasal mucosa), eyelids, and mouth (lips, tongue), and symptoms are generally mild (nasal obstruction, painless discomfort) [14, 18–21].

*L*. *donovani* and *L*. *infantum* are typically responsible for VL, but ML is not so uncommon, especially among immunocompromised subjects (HIV-positive patients, subjects taking corticosteroids or other immunosuppressive drugs) [4, 12, 13]. Patients with ML can present in their clinical history VL [16, 22] or the two clinical forms can coexist and/or rapidly follow each other [12, 42]. Nevertheless, isolated ML cases were frequently reported [4, 12, 13]. Local evolution can be mild and lesions can persist apparently unmodified for years [12, 13]. On the other hand, oral ML (especially by *L*. *donovani*) can cause severe damage, such as tooth loss and severe respiratory obstruction [16]. Lesions are usually described as whitish/reddish/violaceous nodules or polypoid masses, developing in a swollen mucosa. Generally, neoplasia is the main misleading clinical suspicion. Lesions can involve mucous membranes of the nose, cheeks, lips, palate (hard and soft), oropharynx, and larynx [4, 12]. Interestingly, laryngeal involvement can be the only documented presentation of the disease, either in immunocompromised or immunocompetent patients [13]. The most common symptoms are dysphonia, dysphagia, and oral discomfort. Dyspnea can occur when larynx (or trachea) are involved [4, 12, 13].

## 7. Diagnosis

The diagnosis of ML is based on the demonstration of *Leishmania* amastigotes in the mucosal lesions. Histological diagnosis, made on the basis of the observation of amastigotes in mucosal samples stained with Giemsa or hematoxylin-eosin, is probably the most common way to visualize *Leishmania* bodies [21]. However, it seems more effective in Old World than in New World ML. Indeed, in *L*. *infantum *ML histology has good sensitivity (from 50–70% to almost 100%), whereas it decreases to 35–70% in *L*. *braziliensis* ML. Specificity is equally high in both infections (>95%) [6, 10, 12]. The different sensitivity is related to the abundance of *L*. *infantum* bodies in mucosal lesions, whereas *L*. *braziliensis* bodies are moderate or scarce [10, 38]. Culture shows the presence of *Leishmania* promastigotes in medium, such as Novy-McNeal-Nicolle (NNN) and Schneider's liquid [6, 10]. 

Immunoistochemical identification and isoenzyme profiling (multilocus enzyme electrophoresis or MLEE) is useful to confirm the diagnosis and typing of *Leishmania* spp. [4]. MLEE is based on the MON system, which classifies *Leishmania* species according to a combination of 15 enzymes in specific electrophoretic profiles (zymodemes). It primarily targets *L. donovani* complex species [3]. Nowadays, with the diffusion of polymerase chain reaction (PCR), these immunohistochemical and isoenzyme methods appear less adequate than in the past [4].

PCR detects the presence of *Leishmania* DNA/RNA either in culture or tissue specimens [24]. It is a fast method, even effective with low DNA/RNA load, and it works with either amastigote or promastigote DNA/RNA. It is highly sensitive (around 100%) [12]. It amplifies specific sequences of *Leishmania* DNA/RNA by specific primers. The most diffused primers target kDNA (kinetoplast DNA) and SSU-rRNAs (Small SubUnit ribosomal RiboNucleic Acids), such as 18S ribosomal gene, miniexon gene, and ITS1 [3, 6, 12]. Recently, kDNA-PCR was used in the diagnosis of two cases of isolated laryngeal leishmaniasis due to *L*. *infantum* [24]. Among all PCR techniques, RT-PCR (real time-PCR) is expected to achieve the greatest results in the future. Until now, its diffusion has been limited by expensive costs, lack of trained technical operators, and absence of standardized protocols [6, 12]. PCR-ELISA is cheaper than RT-PCR, and for this reason it has been often preferred to RT-PCR [43]. There are evidences about the effectiveness of PCR also when it is applied on cytology brush samples [44]. Sequencing-based pathogen classification and polymorphism-specific PCR recognition are two new interesting applications of PCR methods [3].

As for serological tests, the detection of either anti-*Leishmania* IgG or *Leishmania* antigens can be investigated by several methods. Actually, in *L*. *infantum* ML, the most useful methods are ELISA and immunoblot [12]. In *L*. *braziliensis* MCL immunofluorescence assay (IFA) and ELISA seem to be sensitive (56.7% and 93.3%, resp.). IFA and ELISA are highly sensitive also during *L*. *major* infection [6]. However, all methods are limited by cross-reactions, particularly in New World ML, with antigens of *Trypanosoma cruzi* [6, 10]. Moreover, antigen and antibody blood levels do not reflect real parasite load [3]. Rapid commercials tests detect the presence of *Leishmania* antigens in human samples, giving positive/negative response in five minutes. Recombinant k39 dipstick test is the most commonly used [3, 15]. 

Finally, the Montenegro Skin Test is used in epidemiological studies and, occasionally, for the diagnosis of MCL. It does not distinguish between current and previous infection. It has been reported to have high sensitivity in case of MCL (almost 100%). Probably, it may be useful among travelers to endemic ML areas [6, 10]. 

## 8. Therapy

There are no standardized protocols for the treatment of ML. So, drug choice is based on case reports, clinical experience, and trials. 

Pentavalent antimony (PA) and its derived molecules, sodium stibogluconate (SSB) and meglumine antimoniate (MA), are recommended by World Health Organization (WHO) for the therapy of American MCL [1]. Their killing mechanism is not known, and they are effective only on amastigotes [28]. They are generally used at a dosage of 20 mg/kg/die intravenously for 28 days [1]. For New World *Leishmania* species, their efficacy ranges from 30 to 90% and SSG appears less effective than MA. Their usage is nowadays limited by resistance and toxicity. Resistance is significant in South America, and it is a frequent cause of therapy failure and relapses. It is not clear whether it depends on parasite (natural or acquired) characteristics or on immune defects [1, 28]. On the other hand, these drugs are largely used and effective versus either *L*. *major *or *L*. *infantum* ML [4, 5, 13, 20]. Severe side effects include cardiac (T and ST wave ECG alterations), hepatic (rise of transaminases), haematic (pancytopenia), pancreatic (hyperamylasemia), and renal (glomerulonephritis, hypokalemia) toxicity [45]. The addition of pentoxifylline, a TNF-*α* inhibitor, to PA has been associated with a significant reduction in the healing time in comparison with PA alone for the treatment of ML; in addition, patients treated with pentoxifylline and PA had no need for further courses of treatment, in comparison with 41.6% of patients in the other group [46]. Analogously, the coadministration of pentoxifylline and PA has been associated with a 90% cure rate in patients with ML refractory to PA alone [47].

Pentamidine is highly effective against *Leishmania*, with an efficacy of 90–94% against *L*. *braziliensis*. Unfortunately, resistance is frequent and this is due to loss of intracellular carriers [28]. Recommended dosage is 4 mg/kg/alternate days intravenously (2–4 g total dose), until lesions have disappeared [1]. Its use is markedly limited by severe pancreatic toxicity (diabetes and hypoglycemia), cardiotoxicity (ECG alterations), and renal toxicity (proteinuria) [46]. Because of these severe side effects, WHO recommended to use pentamidine “only when other options are not available” [43].

Amphotericin B (AmB) is an antifungal drug with marked efficacy versus *Leishmania* spp. Amphotericin B binds with ergosterol, a component of fungal and protozoal cellular membrane, forming a transmembrane channel which causes monovalent ion leakage and finally cell death. No resistance has been still reported, but its increasing use for the treatment of leishmaniasis worldwide could potentially enhance the risk to select resistant strains [28]. Cure rates for amphotericin B were similar to those reported with antimonials (88% in a Bolivian study of about 200 patients) [48]. However, renal toxicity thwarts its use as a first-choice drug. Liposomal AmB (LAmB) may be preferred, because it is less nephrotoxic than deoxycolate AmB (DAmB) [1]. In ML, AmB (particularly LAmB) was reported to be effective versus *L. infantum*, *L. donovani* and *L. braziliensis* complex species [1, 4, 12, 13, 28]. WHO recommends a dosage of 2-3 mg/kg/day for 20 days, but successful shorter treatments with higher dosage (3–5 mg/kg/day) have also been reported [1]. Unfortunately, the cost of lipid formulation of amphotericin B is prohibitive in underdeveloped countries.

Miltefosine is highly effective for the treatment of VL. It is orally administrated at 2.5 mg/kg/day for 28 days [1]. It was demonstrated that miltefosine produces apoptosis like-death in *L*. *donovani* promastigotes [3]. Side effects include vomiting, nausea, diarrhea, and hypercreatininaemia [1]. Miltefosine has been successfully used for the treatment of ML due to *L*. *braziliensis*, with cure rates of about 75% in Bolivian patients [49, 50]. 

Paromomycin (or aminosidine) is an aminoglicosyde drug. It interferes with amastigote growth. It is available in topical and intravenous formulations [28]. In VL, intravenous paromomycin is administrated at a dose of 15 mg/kg/day for 21 days [43], whereas in MCL has been reported to have only 60% efficacy (significantly less efficient than PA and AmB) [28]. Successful use of topical paromomycin in *L*. *infantum* ML was described [51]. However, because of the frequent risk of visceral spread during *L*. *infantum* (and *L*. *donovani*) ML, intravenous formulations are preferable [12].

Considering the importance of HIV/*Leishmania* coinfection, it would be interesting to evaluate the role of highly active antiretroviral therapy (HAART) in the outcome of patients with ML. On the one hand, there is clear evidence about benefits of HAART in reducing VL mortality and morbidity; on the other hand, there is a lack of data about contemporary HAART/anti-*Leishmania* therapy in ML. Failure of PA treatment has been reported [9, 37].

## 9. Prevention

As well as in other clinical presentations of leishmaniasis, ML is scarcely suitable for prevention. Theoretically, two approaches are possible: vaccination and territory control [3, 10].

Unfortunately, both these options are still largely unsatisfactory. No effective and safe vaccines are available for humans. The only successful vaccine is active on transmission of canine leishmaniasis. Approaches to anti-*Leishmania* vaccines in humans were made with alive virulent parasites (*L*. *infantum*), killed parasites (*L*. *braziliensis*, *L*. *guyanensis*, and *L*. *amazonensis*) and *Leishmania* proteins (*L*. *major*, *L*. *braziliensis*), with no significant results [3]. 

Territory control is also difficult. Possible strategies include increased surveillance (notification, studies of distribution, and drug resistance), case management (equipped laboratories and access to health care facilities), health education, and research (community-based, sandfly ecology, and risk factor studies) [10].

## 10. Conclusions

Although the majority of cases of ML occur in Latin America, ML is a worldwide disease, whose incidence is expected to rise in the near future as a result of significant environmental and anthroponotic changes. *Leishmania* species of all four complexes may be potential causative agents of ML. Furthermore, an important role is played by vectors, which are largely responsible for the diffusion of the disease and may contribute to the pathogenesis of ML. Diagnosis is actually principally based on histology, but PCR is expected to acquire a major role in the future. Large, randomized trials are needed to define the most effective therapeutic protocol. Prevention perspectives are currently unsatisfactory.
